# Interrupting bedtime to reverse frailty levels in acute care: a study protocol for the Breaking Bad Rest randomized controlled trial

**DOI:** 10.1186/s12877-023-04172-x

**Published:** 2023-08-10

**Authors:** Olga Theou, Myles W. O’Brien, Judith Godin, Chris Blanchard, Leah Cahill, Mohammad Hajizadeh, Peter Hartley, Pamala Jarrett, Dustin Scott Kehler, Roman Romero-Ortuno, Renuka Visvanathan, Kenneth Rockwood

**Affiliations:** 1 School of Physiotherapy, Dalhousie University, Halifax, NS Canada; 2 Division of Geriatric Medicine, Dalhousie University and Nova Scotia Health, Halifax, NS Canada; 3 Department of Medicine, Dalhousie University, Halifax, NS Canada; 4 School of Health Administration, Faculty of Health, Dalhousie University, Halifax, NS Canada; 5 Department of Physiotherapy, Cambridge University Hospital NHS Foundation Trust, Cambridge, UK; 6 Geriatric Medicine, Horizon Health Network, Dalhousie University, Saint John, New Brunswick, Canada; 7 Discipline of Medical Gerontology, School of Medicine, Trinity College Dublin, Dublin, Ireland; 8 Adelaide Geriatrics Training and Research with Aged Care (GTRAC) Centre, School of Medicine, Faculty of Health and Medical Sciences, University of Adelaide, Adelaide, South Australia Australia; 9 Aged and Extended Care Services, The Queen Elizabeth Hospital and Basil Hetzel Institute, Central Adelaide Local Health Network, Adelaide, South Australia Australia

**Keywords:** Frailty, Sedentary time, Step counts, Geriatric medicine, ActivPAL, StepWatch

## Abstract

**Background:**

Hospitalized older patients spend most of the waking hours in bed, even if they can walk independently. Excessive bedrest contributes to the development of frailty and worse hospital outcomes. We describe the study protocol for the **Breaking Bad Rest Study**, a randomized clinical trial aimed to promoting more movement in acute care using a novel device-based approach that could mitigate the impact of too much bedrest on frailty.

**Methods:**

Fifty patients in a geriatric unit will be randomized into an intervention or usual care control group. Both groups will be equipped with an activPAL (a measure of posture) and StepWatch (a measure of step counts) to wear throughout their entire hospital stay to capture their physical activity levels and posture. Frailty will be assessed via a multi-item questionnaire assessing health deficits at admission, weekly for the first month, then monthly thereafter, and at 1-month post-discharge. Secondary measures including geriatric assessments, cognitive function, falls, and hospital re-admissions will be assessed. Mixed models for repeated measures will determine whether daily activity differed between groups, changed over the course of their hospital stay, and impacted frailty levels.

**Discussion:**

This randomized clinical trial will add to the evidence base on addressing frailty in older adults in acute care settings through a devices-based movement intervention. The findings of this trial may inform guidelines for limiting time spent sedentary or in bed during a patient’s stay in geriatric units, with the intention of scaling up this study model to other acute care sites if successful.

**Trial Registration:**

The protocol has been registered at clinicaltrials.gov (identifier: NCT03682523).

**Supplementary Information:**

The online version contains supplementary material available at 10.1186/s12877-023-04172-x.

## Background

Frailty is a state of increased vulnerability to adverse health outcomes, due to the accumulation of health deficits [1]. Hospitalized patients living with frailty have a higher risk for functional decline, new impairments in activities of daily living, a longer hospital stay, hospital readmission, and death [2–4]. The risk of hospital-associated deconditioning may be related to the severity of illness, frailty, and hospital structure/processes of care [5]. In general, hospitalized patients spend > 93% of their time lying in bed awake, even if they can walk independently [6]. This excessive sedentary time puts patients’ in-hospital recovery and post-hospital independence at risk. Sedentary time is characterized by a low energy expenditure (< 1.5 metabolic equivalents of task) during awake hours, while in a seated, reclining, or lying posture [7]. Strategies that help address patients’ excessive time spent in bed may attenuate the accelerated development of frailty that accompanies hospital stays.

Based on thigh-worn inclinometry, hospital inpatients are upright for ~ 50 min/day during their waking hours [8], which is much less than reported values of community dwelling older adults (~ 6 h/day; [9]). Observational data have indicated that older patients who walked at least once/day outside their room during hospitalization had ~ 1.7 days shorter length of hospital stay compared with those who stayed in their room [10]. Greater sedentary time in hospitalised older adults is also associated with greater loss of knee-extension strength [11]. While observational studies support the positive impact of moving more on patient health [8, 10], multiple barriers exist to promoting upright time in a hospital, including fall concerns, pain, and lack of patient motivation [12]. Pragmatic interventional studies are needed to help patients move more and limit their sedentary time to address the health impacts of immobility during a hospital stay.

Frailty is a useful outcome measure of overall health state in acute care and is a predictor of adverse health outcomes [13]. However, few interventional studies have measured frailty as an outcome in hospitalized adults [14]. During a randomized clinical trial, two daily sessions of moderate-intensity exercises improved the frailty levels of hospitalized older adults more than the control group [15]. In another study in acute geriatric care, researchers did not observe changes in frailty between the intervention and control groups in those who did, on average, ~five, ~ 20 min sessions of functional exercise sessions over an average of 18 days hospital stay [16]. Perhaps, more frequent patient contact and/or targeting overall physical activity in the absence of periodic structured exercise but combined with goal setting and feedback on performance may be a more feasible approach to helping inpatients move more. The use of goal setting in hospital has been shown to be independently associated with increased physical activity [17]. Our proposed study will strengthen the evidence base of movement and frailty in healthcare by examining whether a step-count based intervention during a patients’ stay in acute care is successful and reduces the level of frailty during hospitalization. Such an understanding is vital to provide guidance on best evidence practice in reducing excessive bed rest in acute care.

The overarching objective of our ***Breaking Bad Rest*** clinical trial is to provide a safe, feasible, and effective intervention that improves the health of inpatients. Using a novel intervention design that involves frequent patient visits, goal setting, and feedback on performance we will determine the feasibility of our ***Breaking Bad Rest*** intervention model in acute geriatric care. We will test the hypothesis that our intervention will improve physical activity and frailty levels and health outcomes to a greater extent than usual care during hospitalization.

## Methods

### Study design and participant selection

The Breaking Bad Rest clinical trial is a randomized controlled trial. The protocol has been registered at clinicaltrials.gov (identifier: NCT03682523) and a SPIRIT checklist is included as Supplemental File 1.

Participants will be approached for this study within 24 h of their admission to the Geriatric Assessment Unit (GAU). The GAU in the QEII Health Sciences Centre in Halifax, Nova Scotia, Canada is a 14-bed acute care inpatient unit that provides interdisciplinary assessment and treatment to older adults with complex medical and social issues.

Our research team will assess patients for eligibility upon admission. The inclusion criteria for this study are: (1) Anticipated hospital stay of more than one day, and (2) Patient or care partner can communicate in English. The exclusion criteria are: (1) Patient or their care partner are not able to provide informed consent, (2) Bedridden prior to hospital admission, (3) Previous participation in our study (i.e., GAU readmission during data collection phase), (4) Near end-of-life or are waiting for long-term care facility placement at GAU admission, or (5) Patient is admitted to a shared room with a current study participant; to avoid risk of behavioural contamination in shared rooms (e.g., additional motivation or demotivation), only one individual in each room will be recruited into the study. We will describe the study to the prospective participant, and/or that person’s care partner and obtain written informed consent. Participants may request to discontinue the study at any point.

No identifying information will be publicly available; patients will be identified only by their study identification numbers. Access to any file relevant to this study will be limited to the study personnel at Nova Scotia Health Authority (NSHA), the NSHA Research Ethics Board (NSHA-REB) and auditors, upon request.

### Sample size

We will recruit 25 participants for the usual care group and 25 participants for the treatment group. This number is based on a previous observational study in which 62% of individuals had an improvement of ≥ 0.10 in their frailty index score during hospitalization (see below for details) [8]. Using these prior estimates, group sample sizes of 25 per group achieves 80% power to detect a relative increase of 50% (i.e., an increase from 62 to 93%) in the proportion achieving a difference of 0.10 in the frailty index. A increase of 0.10 in the frailty index score has been shown to be associated with various adverse health outcomes including extended length of hospital stay, institutionalization, in-hospital falls, delirium, pressure ulcer incidence, and mortality [18].

### Randomization and blinding

Participants will be randomized into intervention or control by a statistician using an n = 6 block design and stratification by participants’ maximum level of mobility at pre-admission. We will identify the patients’ pre-admission mobility level by asking them or their care partner at admission whether the patient, 1) walked independently (i.e., no walking aid or additional person required), 2) required a walking aid (e.g., walker) but not the assistance of another person, or 3) required the assistance of another person. This blocking and stratified randomization was done using the randomizeR package (v2.0.0) [19] and ensures a close to equal number of participants in each group and that the proportion of patients with the same movement capabilities are distributed evenly into the intervention and control groups. The primary investigator and data collection assessors are blinded to participant group assignment; the data analyst will also be blinded.

### Intervention group

The intervention group will receive usual care, physical activity monitors (see below for details), and visits from researchers to set daily activity goals and promote movement. Specifically, research assistants (with backgrounds in kinesiology) will visit each patient in the morning and afternoon/evening every day during hospitalization (i.e., 14 times per week) to set a step goal in the morning and check on progress towards this goal later in the day. A white board will be positioned in their room and the specific step goal will be written on it as a target for the patient. A target of up to ~ 20% increase in step counts from the previous day will be initially used. If participants have not achieved their daily goal, feedback will be provided to the patients and patients will be safely mobilized to the maximum level of their ability in the afternoon/evening. While there is a specific step goal, this is not designed to be an all or nothing approach, with the patient encouraged to move to the most of their abilities (e.g., those who are sitting a lot will be encouraged to stand more). Patients who do not meet their target will be encouraged to do whatever is possible within their mobility limits. The research assistants will help facilitate movement and offer going for a brief walk if the patient is interested. The overarching purpose is to promote more movement in an acute care setting to facilitate a more realistic adoption of this intervention into clinical care rather than a pass/fail of specific number of steps increased day-to-day.

### Control group

Those randomized into the control group will receive usual care and will undergo the same social engagement as the intervention group but without the encouragement to engage in more activity and setting activity goals. The control group will receive brief visits from the research assistants conducting the intervention at the same time they went into the unit to visit intervention participants to ensure consistency of the social engagement aspect between intervention-control groups. This entails brief (< 5 min) small talk as how their day is going, if the monitor is annoying them, etc. White boards with random numbers will be placed in the control group patients’ rooms so that the researchers responsible for conducting health assessments will be blind to group allocation.

### Measures

**Fig. 1:**
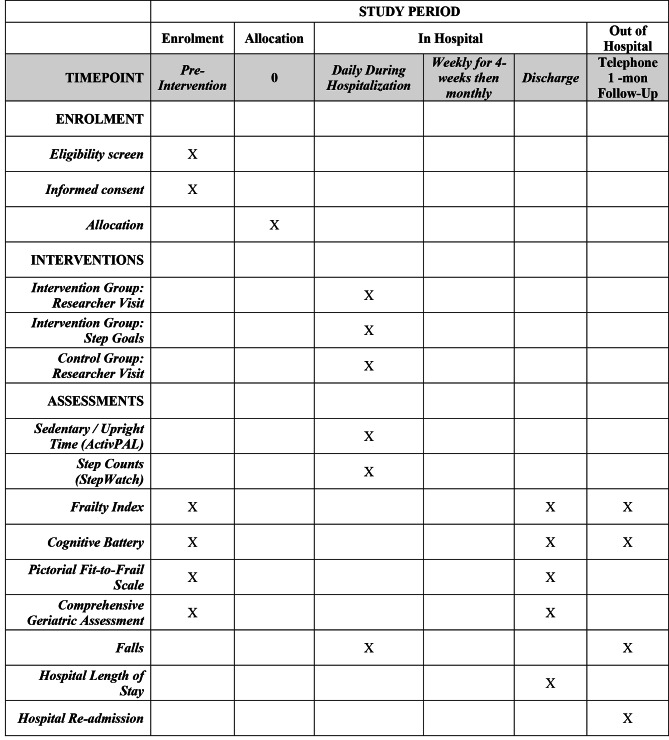
SPIRIT diagram presenting the schedule of enrolment, intervention, and data collection for each research tool over the course of the Breaking Bad Rest trial

The proportion of patients who finish the study, including the within hospital and post-discharge one-month follow-up phone call will be recorded. The schedule for the measurement of primary and secondary outcomes (described in detail below) are presented in Fig. 1. All data will be input into REDCap, a secure web application for surveys and databases, and initially inspected by a member of the research team not involved with the collection of data. Another separate research assistant not involved in conducting the measures will export the data from REDCap and review it for accuracy prior to statistical analyses.

### Physical activity and sedentary time

Physical activity and postural positions will be objectively measured continuously (24-hrs per day for all days) throughout the study for both groups. Within 24-hr of admission to the geriatric unit, patients will be outfitted with an activPAL and StepWatch on their thigh and ankle, respectively. The activPAL (activPAL4, PAL Technologies Ltd) is a valid measure of posture [20], distinguishing time spent in upright postures from sedentary/sleeping postures. This monitor will be waterproofed via a nitrile sleeve and attached using Tegaderm medical dressing and replaced every 14 days, if necessary. The StepWatch (Modus Health) will be positioned around the ankle of participants throughout the duration of their hospital visit. This monitor has demonstrated validity in determining step counts, even at slower stepping speeds [21]. The StepWatch has Bluetooth capabilities and will be used to establish patient step goals throughout the study without being taken off and downloaded. Research assistants download step counts in the morning of each day from the StepWatch and use these values to determine the step goals for that day. The primary activity-related outcomes are step counts (via StepWatch), upright time (via activPAL), and total horizontal time (i.e., sedentary and sleep; via activPAL).

### Frailty index

The frailty index operationalizes frailty in clinical practice and for research [1, 22]. Frailty index scores are calculated as a ratio of health deficits present to total deficits assessed, with a higher value indicating worse frailty levels. For this study, a multi-item frailty index will be calculated based on a health history questionnaire completed by the patient or care partner (if necessary). The specific health questionnaire includes signs/symptoms, mobility, cognition, mood, quality of life, activities of daily living, and nutrition (Supplemental File 2). Frailty will be determined at admission, each week for 1-month (then monthly thereafter), and at a 1-month post-discharge phone call. In hospitalized older adult patients, a change of 0.03 has been demonstrated to be a clinically meaningful change for frailty index scores [23]. In addition to the changes in the continuous frailty score, we will also examine the proportion of patients whose frailty index changes by 0.03 and 0.10 from admission to discharge; 0.10 is considered a moderate change in the frailty index score [24].

### Secondary outcomes

Hospital length of stay will be determined at hospital discharge. Hospital readmissions within 30 days will be quantified by self-report at the one-month follow up time point after hospital discharge. Falls will be tracked by research assistants during their daily visits and will also be assessed by self-report at the one-month follow up time point after hospital discharge.

The Pictorial Fit-to-Frail Scale will also be conducted as a secondary measure of frailty that is completed independently by the patient (or their care partner if necessary) on admission and prior to discharge [25, 26]. A Comprehensive Geriatric Assessment is a multi-disciplinary diagnostic and treatment process that provides a tool to direct patient care. A physician or trained researcher will conduct this Comprehensive Geriatric Assessment upon admission and discharge. A frailty index can be calculated from a Comprehensive Geriatric Assessment and will be quantified in this study [27]. The following cognitive battery tests will be administered at admission, each week for 1-month (then monthly thereafter), and at a 1-month post-discharge phone call: the Rey Auditory Verbal Learning Test (immediate and delayed), Verbal Fluency, Animal Naming, and Mental Alternation Test [28, 29].

### Statistical analysis

Data collected daily (e.g., physical activity outcomes) or weekly/monthly (e.g., frailty index) will be compared between the intervention and the control group using mixed-models for repeated measures. This modelling accounts for the differences in number of observations between participants, with variability in the number of total days of valid physical activity outcomes. A Chi-Square test of independence will be used to compare the proportion of patients who improve their frailty index scores by ≥ 0.10 or ≥ 0.03 in the intervention and control group at hospital discharge. Measurements conducted at admission and discharge only (e.g., comprehensive geriatric assessment) will be examined via a group by timepoint repeated measures or Friedman’s analysis of variance. Other secondary outcomes (e.g., length of hospital) will be analyzed between the intervention and control groups via independent samples *t*-test and Chi-square tests for continuous and categorical outcomes, respectively.

## Status to date

The COVID-19 pandemic has delayed the recruitment of participants from our original start date. This was a particular concern given the increased vulnerability of our population of interest. We have attained Research Ethics Board approval from the Nova Scotia Health (ID: 1023828). As outlined in trial registration (NCT03682523), recruitment for this study is on-going.

## Discussion

The **Breaking Bad Rest** randomized controlled trial aims to promote reducing time in bed and accumulating more step counts during patients’ hospital stay. These findings may provide a novel intervention model for increasing movement in acute care settings and attenuate the development of frailty status among older inpatients. Independent of effectiveness, investigating the feasibility of this type of intervention will be important for the development of future interventions. Specifically, the recruitment of GAU patients that meet our inclusion criteria and conducting a time-intensive intervention protocol to monitor patient activity multiple times each day will be operationally challenging but may inform us if these types of full-time exercise professional positions are warranted. The logistics of timing recruitment within 24-hr of admission to the GAU, conducting weekly assessments in inpatients, and immediately before discharge could be an issue, but the frequent contact of our research team with patients and regular discussions with healthcare providers should mitigate this concern. Importantly, an effort will be put forth to require as little extra work as possible from the GAU staff members (e.g., nurses) to conduct the study, as to test an intervention model that does not add more work responsibilities to the existing staff.

If the study is successful, the model used may be scaled to other acute care units to address the growing concerns of frailty among inpatients.

### Electronic supplementary material

Below is the link to the electronic supplementary material.

Supplementary Material 1

Supplementary Material 2

## Data Availability

Not applicable.
