# Supplementary material for: Interrupting bedtime to reverse frailty levels in acute care: a study protocol for the Breaking Bad Rest randomized controlled trial
Source: BMC Geriatr. 2023 Aug 10;23:482. doi: 10.1186/s12877-023-04172-x (PMC10416381; doi:10.1186/s12877-023-04172-x)
Supplement: Supplementary file 2 — Supplementary Material 2 [file 12877_2023_4172_MOESM2_ESM.docx]

**Supplemental File 2.** Variables included in the frailty index (B = baseline [2 weeks ago or before admission if transferred], A = admission/today)

*1. Chronic conditions*

|  |  | **No** | **Yes** |
| --- | --- | --- | --- |
| Has a doctor ever told you that you … | ... have osteoarthritis in the knee? |  |  |
|  | ... have osteoarthritis in the hip? |  |  |
|  | ... have osteoarthritis in one or both hands? |  |  |
|  | ... have rheumatoid arthritis? |  |  |
|  | .. have any other type of arthritis? |  |  |
|  | …have/had any of the following: emphysema, chronic bronchitis, chronic obstructive pulmonary disease (COPD), or chronic changes in lungs due to smoking? |  |  |
|  | ... have high blood pressure or hypertension? |  |  |
|  | ... have diabetes, borderline diabetes or that your blood sugar is high? |  |  |
|  | ... have heart disease (including congestive heart failure or CHF)? |  |  |
|  | … have angina (or chest pain due to heart disease)? |  |  |
|  | ... have had a heart attack or myocardial infarction? |  |  |
|  | ... have peripheral vascular disease or poor circulation in your limbs? |  |  |
|  | ... have experienced a stroke or CVA (cerebrovascular accident)? |  |  |
|  | ... have experienced a mini-stroke or TIA (transient ischemic attack)? |  |  |
|  | ... have a memory problem? |  |  |
|  | ... have dementia or Alzheimer’s disease? |  |  |
|  | ... had Parkinsonism or Parkinson’s disease? |  |  |
|  | ... have intestinal or stomach ulcers? |  |  |
|  | … have a bowel disorder such as Crohn’s Disease, ulcerative colitis, or Irritable Bowel Syndrome? |  |  |
|  | ... experience bowel incontinence? |  |  |
|  | ... experience urinary incontinence? |  |  |
|  | ... have cataracts? |  |  |
|  | ... have glaucoma? |  |  |
|  | ... have macular degeneration? |  |  |
|  | ... had cancer? |  |  |
|  | ... have osteoporosis, sometimes called low bone mineral density, or thin, brittle or weak bones? |  |  |
|  | ... have back problems, excluding fibromyalgia and arthritis? |  |  |
|  | ... have an UNDER-active thyroid gland (sometimes called hypothyroidism or myxedema)? |  |  |
|  | ... have an OVER-active thyroid gland (sometimes called hyperthyroidism or Graves’ disease)? |  |  |
|  | ... have kidney disease or kidney failure? |  |  |
| In the past year, have you seen a doctor for any of the following reasons? | Pneumonia |  |  |
|  | Urinary tract infection |  |  |

*2. Self-rated weight*

|  | **Just about right** | | **Overweight** | | **Underweight** | |
| --- | --- | --- | --- | --- | --- | --- |
| Do you consider yourself overweight, underweight, or just about right? | B | A | B | A | B | A |

*3.- Overall self-rated health, vision and hearing*

|  | **Excellent** | | **Very good** | | **Good** | | **Fair** | | **Poor**  **or non-existent (i.e. blind)** | |
| --- | --- | --- | --- | --- | --- | --- | --- | --- | --- | --- |
| In general, would you say your health is: | B | A | B | A | B | A | B | A | B | A |
| Is your eyesight, using glasses or corrective lens if you use them: | B | A | B | A | B | A | B | A | B | A |
| Is your hearing, using a hearing aid if you use one: | B | A | B | A | B | A | B | A | B | A |

*4. Falls*

|  | **None** | **Once** | **Two or more times** |
| --- | --- | --- | --- |
| How many times have you fallen in the past year? |  |  |  |
| (if there was a fall in the past 12 months), were any of these falls serious enough to limit some of your normal activities (e.g. broken bone, bad cut or sprain)? |  |  |  |
| (if there was a fall), did any of these falls occur in the past two weeks? |  |  |  |
| (if there was a fall in the past 2 weeks), were any of these falls serious enough to limit some of your normal activities (e.g. broken bone, bad cut or sprain)? |  |  |  |
| (at discharge) how many times did you fall while in hospital? |  |  |  |
| (if there was a fall during hospitalisation), were any of these falls serious enough to limit some of your normal activities (e.g. broken bone, bad cut or sprain)? |  |  |  |

*5. Mood-related items*

|  | | **Rarely or never (less than 1 day)** | | **Some of the time**  **(1-2 days)** | | **Occasionally (3-4 days)** | | **All of the time**  **(5-7 days)** | |
| --- | --- | --- | --- | --- | --- | --- | --- | --- | --- |
| How often did you feel… | …that everything you did was an effort? | B | A | B | A | B | A | B | A |
|  | …lonely? | B | A | B | A | B | A | B | A |
|  | …that you could not “get going”? | B | A | B | A | B | A | B | A |
|  | Sad or depressed | B | A | B | A | B | A | B | A |

*6. Mobility*

|  |  | **No** | | **Yes, the difficulty degree is…** | | | | | | **Can´t do it** | | **Don’t do**  **on doctor’s orders** | |
| --- | --- | --- | --- | --- | --- | --- | --- | --- | --- | --- | --- | --- | --- |
|  |  |  |  | **A little** | | **Somewhat** | | **Very** | |  |  |  |  |
| Do you have any difficulty | … reaching or extending your arms above your shoulders? | B | A | B | A | B | A | B | A | B | A | B | A |
|  | …stooping, crouching, or kneeling down? | B | A | B | A | B | A | B | A | B | A | B | A |
|  | …pushing or pulling large objects like a living room chair? | B | A | B | A | B | A | B | A | B | A | B | A |
|  | …lifting 10 pounds (or 4.5kg) from the floor, like a heavy bag of groceries? | B | A | B | A | B | A | B | A | B | A | B | A |
|  | …handling small objects, like picking up a coin from a table? | B | A | B | A | B | A | B | A | B | A | B | A |
|  | …standing for a long period, around 15 minutes? | B | A | B | A | B | A | B | A | B | A | B | A |
|  | …standing up after sitting in a chair? | B | A | B | A | B | A | B | A | B | A | B | A |
|  | …walking alone up and down a flight of stairs? | B | A | B | A | B | A | B | A | B | A | B | A |
|  | …walking 2 to 3 neighbourhood blocks? | B | A | B | A | B | A | B | A | B | A | B | A |
|  | …making a bed? | B | A | B | A | B | A | B | A | B | A | B | A |
|  | …washing your back? | B | A | B | A | B | A | B | A | B | A | B | A |
|  | …using a knife to cut food? | B | A | B | A | B | A | B | A | B | A | B | A |
|  | …with recreational or work activities in which you take some force or impact through your arm, shoulder, or hand (e.g., golf, hammering, tennis, typing)? | B | A | B | A | B | A | B | A | B | A | B | A |

*7. Activities of daily living (ADL) and instrumental activities of daily living (IADL)*

|  |  | **Yes** | | **No** | | **Don’t know/**  **No Answer** |
| --- | --- | --- | --- | --- | --- | --- |
| Can you…  Can you… | …dress and undress yourself without help (e.g. picking out clothes and putting on socks & shoes)? | B | A | B | A | (Skip to next ADL) |
|  |  | (Skip to next ADL) | | (Continue questioning) | |  |
|  | (if no) …dress and undress yourself with some help? | B | A | B | A |  |
|  |  | (Skip to next ADL) | | (Continue questioning) | |  |
|  | (if no) Are you completely unable to dress and undress yourself? | B | A | B | A |  |

|  |  | **Yes** | | **No** | | **Don’t know/**  **No Answer** |
| --- | --- | --- | --- | --- | --- | --- |
| Can you…  Can you… | …Can you take care of your own appearance without help (e.g. combing your hair, or  shaving? | B | A | B | A | (Skip to next ADL) |
|  |  | (Skip to next ADL) | | (Continue questioning) | |  |
|  | (if no) …take care of your own appearance with some help? | B | A | B | A |  |
|  |  | (Skip to next ADL) | | (Continue questioning) | |  |
|  | (if no) Are you completely unable to take care of your own appearance? | B | A | B | A |  |

|  |  | **Yes** | | **No** | | **Don’t know/**  **No Answer** |
| --- | --- | --- | --- | --- | --- | --- |
| Can you…  Can you… | …walk without help? | B | A | B | A | (Skip to next ADL) |
|  |  | (Skip to next ADL) | | (Continue questioning) | |  |
|  | (if no) …walk with some help from a person? | B | A | B | A |  |
|  |  | (Skip to next ADL) | | (Continue questioning) | |  |
|  | (if no) …walk with the use of a walker or crutches, etc.? | B | A | B | A |  |
|  |  | (Skip to next ADL) | | (Continue questioning) | |  |
|  | (if no) Are you completely unable to walk? | B | A | B | A |  |

|  |  | **Yes** | | **No** | | **Don’t know/**  **No answer** |
| --- | --- | --- | --- | --- | --- | --- |
| Can you…  Can you… | …get in and out of bed without any help or aids? | B | A | B | A | (Skip to the next ADL) |
|  |  | (Skip to next ADL) | | (Continue questioning) | |  |
|  | (if no) …get in and out of bed with some help (either from a person or with the aid of some device)? | B | A | B | A |  |
|  |  | (Skip to next ADL) | | (Continue questioning) | |  |
|  | (if no) Are you totally dependent on someone else to lift you in and out of bed? | B | A | B | A |  |

|  |  | **Yes** | | **No** | | **Don’t know/**  **No answer** |
| --- | --- | --- | --- | --- | --- | --- |
| Can you…  Can you… | …take a bath or shower without help? | B | A | B | A | (Skip to IADLs) |
|  |  | (Skip to next ADL) | | (Continue questioning) | |  |
|  | (if no) …take a bath or shower with some help (i.e. you need help from someone getting in and out of the tub or you need special attachments on the tub)? | B | A | B | A |  |
|  |  | (Skip to next ADL) | | (Continue questioning) | |  |
|  | (if no) Are you completely unable to take a bath or shower by yourself? | B | A | B | A |  |

|  |  | **Yes** | | **No** | | **Don’t know/**  **No answer** |
| --- | --- | --- | --- | --- | --- | --- |
| Can you…  Can you… | …use the telephone without help, including looking up numbers and dialling? | B | A | B | A | (Skip to the next IADL) |
|  |  | (Skip to next IADL) | | (Continue questioning) | |  |
|  | (if no) …use the telephone with some help (i.e., you can answer the phone or dial the operator in an emergency, but need a special phone or help in getting the number or dialing)? | B | A | B | A |  |
|  |  | (Skip to next IADL) | | (Continue questioning) | |  |
|  | (if no) Are you completely unable to use the telephone? | B | A | B | A |  |

|  |  | **Yes** | | **No** | | **Don’t know/**  **No answer** |
| --- | --- | --- | --- | --- | --- | --- |
| Can you…  Can you… | …get to places out of walking distance without help (i.e., you drive your own car, or travel alone on buses, or taxis)? | B | A | B | A | (Skip to the next IADL) |
|  |  | (Skip to next IADL) | | (Continue questioning) | |  |
|  | (if no) …get to places out of walking distance with some help (i.e., you need someone to help you or go with you when travelling)? | B | A | B | A |  |
|  |  | (Skip to next IADL) | | (Continue questioning) | |  |
|  | (if no) Are you unable to travel unless emergency arrangements are made for a specialized vehicle, like an ambulance? | B | A | B | A |  |

|  |  | **Yes** | | **No** | | **Don’t know/**  **No answer** |
| --- | --- | --- | --- | --- | --- | --- |
| Can you…  Can you… | …go shopping for groceries or clothes without help (i.e., taking care of all shopping needs yourself)? | B | A | B | A | (Skip to the next IADL) |
|  |  | (Skip to next IADL) | | (Continue questioning) | |  |
|  | (if no) …go shopping for groceries or clothes with some help (i.e., you need someone to go with you on all shopping trips)? | B | A | B | A |  |
|  |  | (Skip to next IADL) | | (Continue questioning) | |  |
|  | (if no) Are you completely unable to do any shopping? | B | A | B | A |  |

|  |  | **Yes** | | **No** | | **Don’t know/**  **No answer** |
| --- | --- | --- | --- | --- | --- | --- |
| Can you…  Can you… | …prepare your own meals without help (i.e., you plan and cook full meals yourself)? | B | A | B | A | (Skip to the next IADL) |
|  |  | (Skip to next IADL) | | (Continue questioning) | |  |
|  | (if no) …prepare your own meals with some help (i.e., you can prepare some things but are unable to cook full meals yourself)? | B | A | B | A |  |
|  |  | (Skip to next IADL) | | (Continue questioning) | |  |
|  | (if no) Are you completely unable to prepare any meals? | B | A | B | A |  |

|  |  | **Yes** | | **No** | | **Don’t know/**  **No answer** |
| --- | --- | --- | --- | --- | --- | --- |
| Can you…  Can you… | …do your housework without help (i.e., you can clean floors, etc.)? | B | A | B | A | (Skip to the next IADL) |
|  |  | (Skip to next IADL) | | (Continue questioning) | |  |
|  | (if no) …do your housework with some help (i.e., you can do light housework but need help with heavy work)? | B | A | B | A |  |
|  |  | (Skip to next IADL) | | (Continue questioning) | |  |
|  | (if no) Are you completely unable to do any housework? | B | A | B | A |  |

|  |  | **Yes** | | **No** | | **Don’t know/**  **No answer** |
| --- | --- | --- | --- | --- | --- | --- |
| Can you…  Can you… | …take your own medicine without help (in the right doses at the right time)? | B | A | B | A | (Skip to the next IADL) |
|  |  | (Skip to next IADL) | | (Continue questioning) | |  |
|  | (if no) …take your own medicine with some help (i.e., you are able to take medicine if someone prepares it for you or reminds you to take it)? | B | A | B | A |  |
|  |  | (Skip to next IADL) | | (Continue questioning) | |  |
|  | (if no) Are you completely unable to take your medicine? | B | A | B | A |  |

|  |  | **Yes** | | **No** | | **Don’t know/**  **No answer** |
| --- | --- | --- | --- | --- | --- | --- |
| Can you…  Can you… | …handle your own money without help (i.e., you write cheques, pay bills, etc.)? | B | A | B | A | (Skip to the next question) |
|  |  | (Skip to next IADL) | | (Continue questioning) | |  |
|  | (if no) …handle your own money with some help (i.e., you manage day-to-day buying but need help with managing your chequebook or paying your bills)? | B | A | B | A |  |
|  |  | (Skip to next IADL) | | (Continue questioning) | |  |
|  | (if no) Are you completely unable to handle your money? | B | A | B | A |  |

| *8. Symptoms* | | **Yes** | | **No** | |
| --- | --- | --- | --- | --- | --- |
| In the past week, have you been bothered by the following… (No=0; Yes= 1) | … Pain in your back, knees, hips, or other joint | B | A | B | A |
|  | …heart trouble or angina, chest pain | B | A | B | A |
|  | …Breathlessness, difficulty breathing | B | A | B | A |
|  | …Sore throat | B | A | B | A |
|  | …Swollen joints | B | A | B | A |
|  | …A fall | B | A | B | A |
|  | …A fall serious enough to limit some of your normal activities (e.g. broken bone, bad cut or sprain) | B | A | B | A |
|  | …Fear of falling down | B | A | B | A |
|  | …Dizziness, light-headedness faints or blackouts | B | A | B | A |
|  | …Stomach or intestine problems, including constipation, gas, diarrhea | B | A | B | A |
|  | …Incontinence or involuntary loss of urine | B | A | B | A |
|  | …Fatigue | B | A | B | A |
|  | …Numbness or tingling in arms or legs | B | A | B | A |
|  | …Muscle spasms | B | A | B | A |
|  | …Muscle weakness | B | A | B | A |
|  | …Sleeping problems, including problems falling asleep, staying asleep, getting back to sleep | B | A | B | A |
|  | …Vivid dreams | B | A | B | A |
|  | …Vivid dreams while awake (hallucinations) | B | A | B | A |
|  | …Sleepy during day | B | A | B | A |
|  | …Trouble concentrating/Brain fog | B | A | B | A |
|  | …Confusion | B | A | B | A |
|  | …Heartburn or Indigestion | B | A | B | A |
|  | …Nausea | B | A | B | A |
|  | …Loss of appetite | B | A | B | A |
|  | …Headache | B | A | B | A |
|  | …Skin is sensitive/Irritated skin | B | A | B | A |
|  | …Itching | B | A | B | A |
|  | …Ringing in the ears | B | A | B | A |
|  | …Eye pain/dryness | B | A | B | A |
|  | …Anxious or Stressed out | B | A | B | A |
|  | …Easily irritated | B | A | B | A |
|  | …Anger or Hostility | B | A | B | A |

*9. Appetite*

|  | **No less desire for food than usual** | | **Less desire for food than usual** | |
| --- | --- | --- | --- | --- |
| How was your appetite? | B | A | B | A |

*10. Walking Aid*

|  |  | **Yes** | | **No** | |
| --- | --- | --- | --- | --- | --- |
| If you have been out of bed, did you use any of the following items… | None | B | A | B | A |
|  | A cane or walking stick | B | A | B | A |
|  | A zimmer frame or walker | B | A | B | A |
|  | Manual, electric wheelchair or buggy/scooter | B | A | B | A |

*11. Self-rated health on a 0-100 scale*

| Self-rated health | B | A |
| --- | --- | --- |
|  | 0-100 Score | 0-100 Score |
